# Self-Propelled Aero-GaN Based Liquid Marbles Exhibiting Pulsed Rotation on the Water Surface

**DOI:** 10.3390/ma14175086

**Published:** 2021-09-06

**Authors:** Tudor Braniste, Vladimir Ciobanu, Fabian Schütt, Hidenori Mimura, Simion Raevschi, Rainer Adelung, Nicola M. Pugno, Ion Tiginyanu

**Affiliations:** 1 National Center for Materials Study and Testing, Technical University of Moldova, Stefan cel Mare Av. 168, 2004 Chisinau, Moldova; tudor.braniste@cnstm.utm.md (T.B.); vladimir.ciobanu@cnstm.utm.md (V.C.); 2 Institute for Materials Science, Kiel University, Kaiserstr. 2, 24143 Kiel, Germany; fas@tf.uni-kiel.de (F.S.); ra@tf.uni-kiel.de (R.A.); 3 Research Institute of Electronics, Shizuoka University, 3-5-1 Johoku, Naka-ku, Hamamatsu 432-8011, Japan; mimura.hidenori@shizuoka.ac.jp; 4 Department of Physics and Engineering, State University of Moldova, Alexei Mateevici Str. 60, 2009 Chisinau, Moldova; raevskis@mail.ru; 5 Laboratory for Bioinspired, Bionic, Nano, Meta Materials & Mechanics, Department of Civil, Environmental and Mechanical Engineering, University of Trento, 38123 Trento, Italy; nicola.pugno@unitn.it; 6 School of Engineering and Materials Science, Queen Mary University of London, Mile End Road, London E1 4NS, UK; 7 Academy of Sciences of Moldova, Stefan cel Mare Av. 1, 2001 Chisinau, Moldova

**Keywords:** aerogalnite, aero-GaN, liquid marble, pulsed rotation

## Abstract

We report on self-propelled rotating liquid marbles fabricated using droplets of alcoholic solution encapsulated in hollow microtetrapods of GaN with hydrophilic free ends of their arms and hydrophobic lateral walls. Apart from stationary rotation, elongated-spheroid-like liquid marbles were found, for the first time, to exhibit pulsed rotation on water surfaces characterized by a threshold speed of rotation, which increased with the weight of the liquid marble while the frequency of pulses proved to decrease. To throw light upon the unusual behavior of the developed self-propelled liquid marbles, we propose a model which takes into account skimming of the liquid marbles over the water surface similar to that inherent to flying water lily beetle and the so-called helicopter effect, causing a liquid marble to rise above the level of the water surface when rotating.

## 1. Introduction

Liquid marbles, discovered by Aussillous and Quéré in 2001 [1], represent aggregates composed of a droplet of liquid encased in and stabilized by a shell of nano- and/or microparticles which, in most cases, possess hydrophobic properties. Honeydew droplets coated by powdery hydrophobic wax secreted by aphids are considered as natural analogous of liquid marbles (LM) [2]. Among specific characteristics inherent to liquid marbles which attracted increasing attention of the scientific community, one can mention permeability of their shell to gases, elasticity, stability on solid and liquid surfaces, along with non-wetting behavior and the ability to non-stick on solid surfaces. Liquid marbles, also known as “dry waters”, demonstrated huge potential for use in microfluidics for controlled transport and release of the small quantities of liquids as well as in sensorics [3,4], microrobotics [5,6], biomedicine [7,8], etc. In particular, Han et al. [9,10] reported on precise control over mass transportation and distribution in the droplet of the liquid marbles synchronously rotating with an external magnetic field, thus opening opportunities for the development of various micromagneto-mechanical devices for use in microfluidics.

In the last decade, research efforts have been undertaken to develop self-propelled liquid marbles exhibiting translational motion, rotation or their combination, self-propulsion being reached by adding volatile substances to the core liquid. In particular, self-propelled liquid marbles have been fabricated using droplets of aqueous ethanol solutions encapsulated by loose polytetrafluoroethylene particles with 1 µm diameter [11] or by fumed fluorosilica powder consisting of 20–30 nm diameter particles [12]. The occurrence of self-propulsion is attributed to the Marangoni solutocapillary flow cropping up when a gradient of surface tension of the fluid support is generated in the neighboring surrounding area of the liquid marble. The gradient can be induced by simply breaking the spherical symmetry of the marbles. In such a case, the evaporation of alcohol and its condensation on the surrounding fluid surface as well as the resulting decrease in the surface tension prove to be spherically asymmetric, thus giving rise to the solutocapillary effect.

Over the last years, we developed wide-band-gap-semiconductor-compound-based aero-materials consisting of hollow microtetrapods, which prove to be promising for a variety of applications [13,14,15,16,17,18,19]. Furthermore, we demonstrated self-propelled, asymmetrically shaped aero-GaN and aero-ZnS based liquid marbles with the liquid droplet composed of alcoholic solution [13,14]. The liquid marbles manifested self-propelled rotational motion, attaining speeds as high as 12.5 and 0.6 rot/s for aero-GaN and aero-ZnS, respectively. In this work, we report in premiere on self-propelled aero-GaN liquid marbles exhibiting pulsed rotation on the water surface. Results of analytical and numerical modeling are presented to account for the observed phenomena.

## 2. Materials and Methods

### 2.1. Aero-GaN Preparation

The Aero-GaN nanomaterial was produced by depositing a thin GaN layer on sacrificial templates composed of highly porous ZnO networks of microtetrapods [13]. The ZnO templates were obtained using the flame transport synthesis approach, as previously described in ref. [20]. For GaN growth, a hydride vapor phase epitaxy (HVPE) system equipped with a horizontal reactor was used. At the first stage, metallic gallium interacts with gaseous hydrogen chloride at 850 °C, resulting in the formation of gallium chloride (GaCl). The GaCl and NH_3_ gas reacted with each other in the next reaction zone, where at the beginning, the temperature was kept at 600 °C for 10 min to initiate nucleation of GaN on the surface of ZnO microtetrapods, and then increased up to *T*_g_ = 850 °C for other 10 min to produce the high quality GaN layer. The flow rates of HCl (15 sml/min), NH_3_ (600 sml/min) and H_2_ (3600 sml/min) were maintained constant during the growth process.

**Figure 1:**
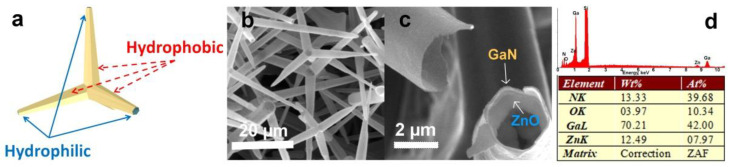
(**a**) Schematic representation of a single Aero-GaN microtetrapod exhibiting both hydrophobic and hydrophilic properties; (**b**) an SEM picture taken from a network of Aero-GaN microtetrapods; (**c**) cross-sectional views of individual microtubes, whose chemical compositions are presented in (**d**).

As demonstrated recently [13], during the process of GaN growth, simultaneous gradual decomposition and removal of the underneath ZnO template occurs due to harsh reaction conditions and high temperatures, thus resulting in the formation of hollow micro-tetrapods with the wall thickness of 10–15 nm. It is to be noted, however, that ZnO is not completely removed and its traces, with the amount of 7 at%, are found on the inner surface of GaN hollow micro-tetrapods. The ZnO traces can be reduced down to 0.7 at% by subjecting the aero-GaN specimens to additional treatment in hydrogen atmosphere at a temperature as high as 900 °C [13]. Thus, according to the results of previous investigations, the aero-GaN consists of networks of interpenetrated GaN hollow micro-tetrapods, the inner surface of which is covered by an ultrathin film of zinc oxide. Note that the outer surface of tetrapod arms is superhydrophobic, with the exception of their free ends, which exhibit superhydrophilicity (Figure 1), thus leading to the occurrence of dual hydrophobic/hydrophilic properties [13]. Figure 1 shows the morphology of the building blocks (aero-tetrapods of GaN) forming the outer layer of the liquid marbles. The schematic representation of a single tetrapod is presented in Figure 1a, which indicates the superhydrophobic and superhydrophilic parts. A network of interpenetrated microtetrapods is presented in Figure 1b, where one can notice individual hollow microtetrapods. The broken arm of a microtetrapod is presented in Figure 1c, demonstrating the tubular structure with nanoscale thickness of the walls. The chemical composition of the structure is presented in Figure 1d, which highlights the presence of an ultrathin layer of ZnO on the inner side of the GaN microtubes, as demonstrated previously [13].

### 2.2. Aero-GaN Liquid Marbles Formation

Liquid marbles were fabricated by wrapping aqua solution droplets in aero-GaN structures. First, the liquid microdroplets were strolled on the surface of GaN aerotetrapods, which were randomly trapped on the droplet surface until a homogeneous porous mantle was built. Next, a part of the liquid marble was subjected to mechanical deformation with the aim to deviate from the spherical symmetry towards an elongated-spheroid-like shape, which, according to previous investigations, is beneficial for the occurrence of self-propelled rotations [13]. The solution used to propel the marbles consisted of commercially available alcoholic solution with ingredients enabling to maintain the surface tension. The pulsed rotation of the LM was investigated using a high-speed camera, Sony FDR-AX700. The angular velocity was calculated by processing the video files.

## 3. Results and Discussions

**Figure 2:**
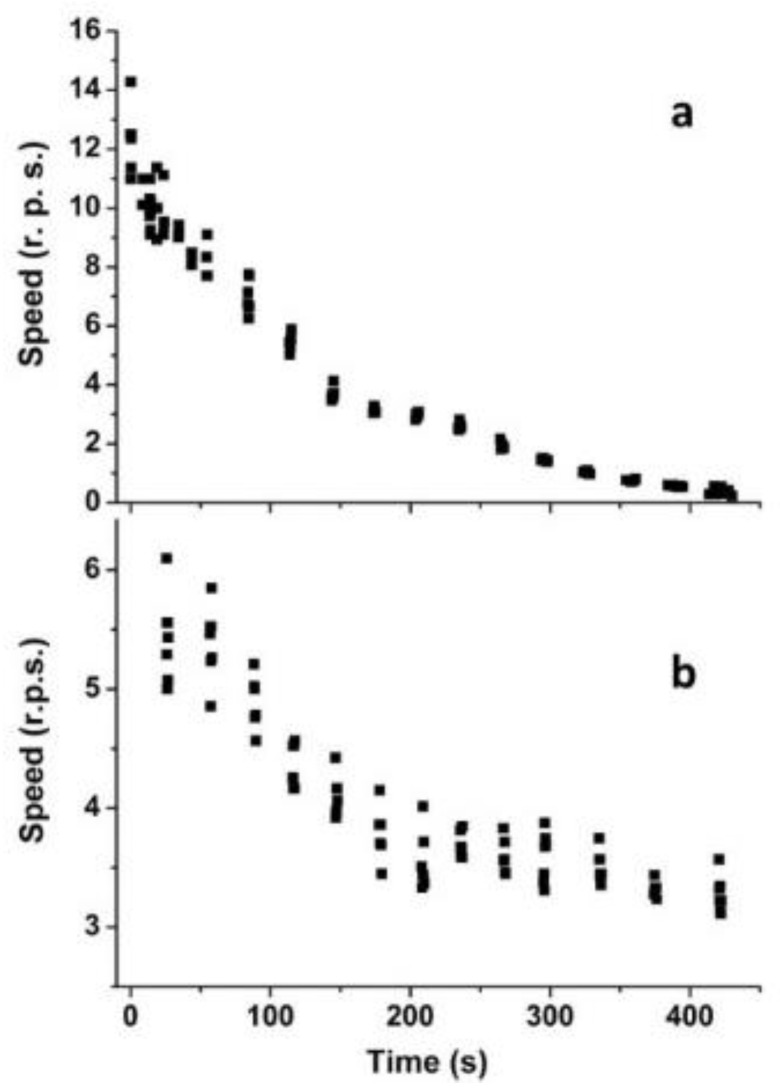
Time dependence of the speed (r.p.s.—rotation per second) of uniform rotation for liquid marbles with different weights: (**a**) 2.5 mg; (**b**) 59.5 mg.

Figure 2 illustrates the time dependence of the speed of uniform rotation for two liquid marbles with weights of 2.5 and 59.5 mg. One can see that the speed of rotation decreases in time in both cases; however, there are important differences in the behavior of the two marbles. First, a lighter liquid marble shows at the beginning a more than two times higher speed of rotation than the heavier one. Second, the rotation of a lighter liquid marble practically ceases in about seven minutes, while the heavier marble is characterized by a rotation with a much higher inertia, namely, the speed of rotation decreases by only 1.7 times in the same interval of time.

As explained in Ref. [13], the speed of rotation as high as 12.5 rot/s attained by the aerogalnite-based liquid marbles is attributed to the specific architecture of the hollow tetrapods enveloping the marble and to the lucky combination of the superhydrophobic and superhydrophilic properties. Since only the free ends of the hollow tetrapod arms pierce through the water surface, between the aero-GaN shell and the water surface, there is a layer of air crossed by a superhydrophobic network. Under these conditions, the surface tension pins the air–water interface to the free ends of the tetrapod arms. Upon rotation of the liquid marble, the free arm ends touching the water glide over its surface, which results in a negligible water drag, thus allowing highly energy efficient motion [13]. The lighter liquid marble loses the speed of rotation faster than the heavier one due to the smaller amount of the volatile compounds in the core droplet and their enhanced evaporation under conditions of circular hydrodynamic flow and outward centrifugal force emerging in the process of fast rotation [9].

**Figure 3:**
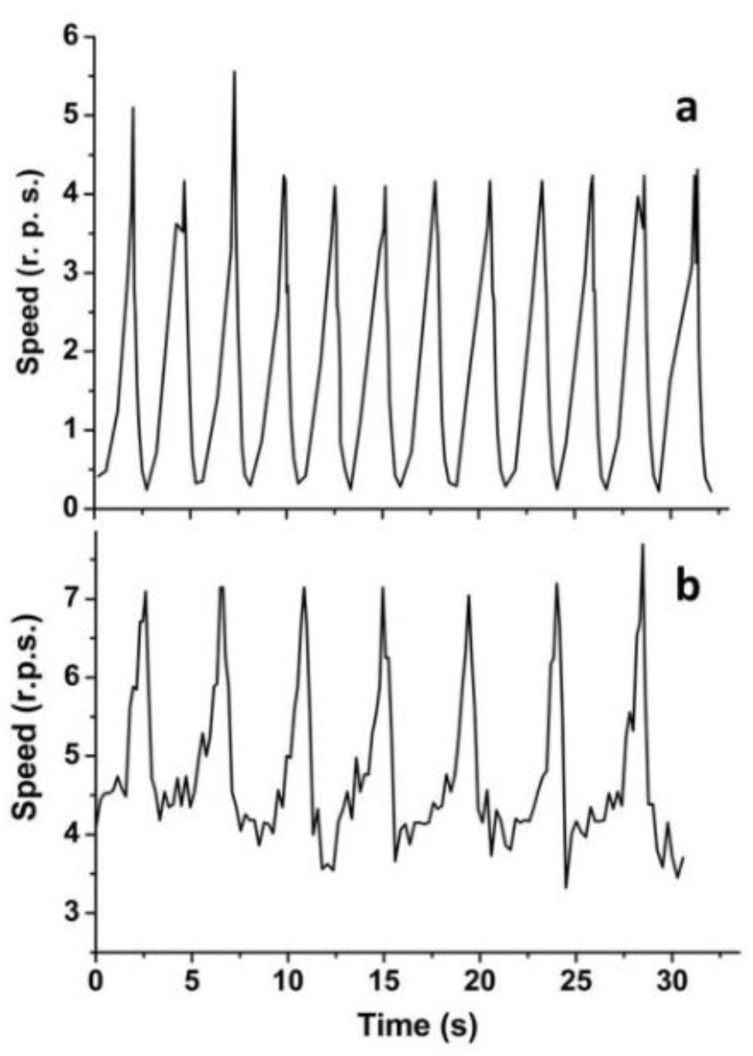
Time dependence of the speed of pulsed rotation for liquid marbles with different weights: (**a**) 6.5 mg; (**b**) 14.5 mg.

Some of the relatively light liquid marbles with spheroid-like shape were found to exhibit pulsed rotation, as illustrated in Figure 3 for liquid marbles with a weight of 6.5 and 14.5 mg. Careful analysis of the experimental results emphasized two important features. First, the speed of rotation is oscillating periodically, the period of oscillation being smaller for the lighter liquid marble. Second, within a definite period of rotation, the speed increases until it reaches a threshold value, followed by a relatively sharp decrease in rotational speed. Note that the threshold speed is higher in the case of a heavier liquid marble (Figure 3b).

**Figure 4:**
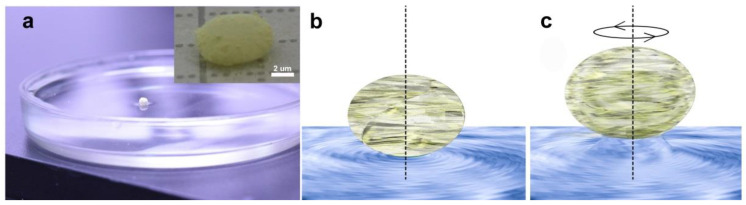
(**a**) Liquid marble floating on water, where the inset represents a digital picture of an elongated liquid marble. (**b**) Schematic interpretation of the liquid marble on the water surface; (**c**) the same liquid marble when rotating at high velocities, leading to the formation of a water column.

To throw light upon the observed fascinating behavior of liquid marbles rotating on a liquid surface, we propose the so-called helicopter-like mechanism of self-propulsion [21]. It takes into account the specific architecture of the GaN hollow tetrapods constituting the shell of the liquid marble. Imagine that the aerogalnite-based shell consists of one monolayer of GaN hollow microtetrapods. In this case three arms of each tetrapod will keep the microtetrapod floating on the core liquid surface, while the fourth one will be among the arms of tetrapods that touch the external water surface, thus ensuring an energy-efficient rotational motion, or among arms positioned totally in air. A floating liquid marble on a water surface is presented in Figure 4a. When the liquid marble rotates, the arms positioned totally in air may play the role of helicopter blades, leading to the generation of the lift force. However, breaking from the water surface is not possible because the ends of free tetrapod arms are hydrophilic and attract water. Under these conditions, the liquid marbles are able to skim over the water surface, similar to the flying water lily beetle [22,23], while any attempt of breaking liquid marble from the water surface will lead to the formation of water microcolumns attached to the ends of free tetrapod arms which, in its turn, will sharply inhibit the rotational process. A schematic representation of a water column under a rotating liquid marble is shown in Figure 4c, which is correlated to the previously reported observations of the water pillars formed when an Aero-GaN sample is being lifted up with a charged amber stick (Figure 5 from the ref. [13]). From general considerations, the lift force will be generated at a definite speed of rotation, which in our experiments is the threshold speed. Obviously, the threshold speed should depend upon the weight of the marble: the lighter the marble, the lower the threshold speed.

This behavior can be better understood using a simple analytical model. The liquid marble is assumed as cylindrical with a radius *r* and height *h*. The resistant torque during rotation in air is *C*_r_ = 2π*rhr*τ, where τ = μd*v*/d*r* = μω is the shear stress acting on its lateral surface, μ is the air viscosity, *v* and ω are the linear and angular velocity and, thus, *C*_r_ = 2π*r*^2^*h*μω = cω. The driving torque *C*_m_ can be modeled according to the discussed helicopter effect, assuming d*C*_m_/dt = k(ω_0_ − ω), so as to have a linear decrement of its rate, where k and ω_0_ are two constants. Imposing the dynamic equilibrium of the marble, we find *Q*d^2^ω/dt^2^ + cdω/dt + kω = kω_0_, where *Q* is the moment of inertia of the marble. In the experiments, no significant viscosity/damping effects are observed. Accordingly, the solution for c ≈ 0 is ω ≈ ω_0_ + AcosΩt + BsinΩt, where the fundamental angular frequency of the oscillations is predicted to be Ω = (k/*Q*)^1/2^. The moment of inertia *Q* scales as M^5/3^, where M is the marble mass (the moment of inertia scales as R^5^, with R being the characteristic size, while M scales as R^3^). The constant k does not have a clear scaling, but two limiting conditions could be envisioned: a negligible scaling, i.e., M^0^, or as proportional to c/t (i.e., *C*_r_ scales, such as *C*_m_) and, thus, as M^1^. As a result, a scaling of the period T = 2π/Ω∝M^5/12–5/6^ = M^0.42–0.83^ is theoretically predicted, whereas we observe experimentally (from only the two available experiments; thus, this comparison has to be considered with caution) T∝M^0.60^, in agreement with this simple model. Fixing the origin of the time reference system at the stationary point for ω implies dω/dt(0) = 0, and thus, B = 0; accordingly, ω_0_ represents the mean value of the angular velocity and A = Δω the amplitude of its oscillation, i.e., ω ≈ ω_0_ + Δω·cosΩt.

## 4. Conclusions

Self-propelled elongated spheroid-like liquid marbles were found for the first time to exhibit pulsed rotation. The maximum speed of rotation attained in each pulse or, in other words, the threshold speed of rotation, increases with the weight of the elongated aero-GaN based liquid marbles. At the same time, the period of pulsed rotation decreases with the weight of the marbles, which is explained using a simple analytical model. For the stationary rotation, we found that both the braking speed and the maximum rotational speed inherent to the beginning of the process decrease with the liquid marble weight. The obtained results pave the way to the development of various self-propelled rotating liquid marbles, in particular for the advancement of micro biological reactors that are capable to host living cells, which are separated by the outer media through an ultra-porous membrane with controlled properties, allowing further advancements in the study of living cells in specific spatially-confined conditions [24].

## Data Availability

The data presented in this study are available on request from the corresponding author.
